# Mesenchymal stem cell-based therapy for radiation-induced lung injury

**DOI:** 10.1186/s13287-018-0776-6

**Published:** 2018-01-31

**Authors:** Tiankai Xu, Yuyu Zhang, Pengyu Chang, Shouliang Gong, Lihong Shao, Lihua Dong

**Affiliations:** 1 Department of Radiation Oncology, The First Bethune Hospital of Jilin University, Changchun, Jilin 130021 China; 2 Key Laboratory of Radiobiology, Ministry of health, School of Public Health, Jilin University, Changchun, 130021 China

**Keywords:** Mesenchymal stem cells (MSCs), Radiation-induced lung injury (RILI), Stem cell therapy

## Abstract

Since radiotherapy is widely used in managing thoracic tumors, physicians have begun to realize that radiation-induced lung injury (RILI) seriously limits the effects of radiotherapy. Unfortunately, there are still no effective methods for controlling RILI. Over the last few decades numerous studies have reported the beneficial effects of mesenchymal stem cells (MSCs) on tissue repair and regeneration. MSCs can not only differentiate into lung alveolar epithelial cells and secrete anti-inflammatory factors, but they also deliver some vehicles for gene therapy in repairing the injured lung, which provides new ideas for managing RILI. Thus, many scientists have attempted to manage RILI using MSC-based therapy. However, as a novel therapy MSCs still face various limitations. Herein, we shed light on the current understanding of MSC-based therapy for RILI, including the feasibility, molecular mechanisms, animal studies, and clinical research of MSC-based therapy for RILI. We also present an overview of RILI and MSCs.

## Background

Over the last few decades, radiotherapy has become one of the most important treatment modalities for thoracic tumors [1]. Even though more localized dose delivery to patients with tumors via advanced radiation techniques can increase the survival rate and lessen radiation-related toxicity, the occurrence of radiation-induced lung injury (RILI) is still inevitable and limits dose escalation for thoracic radiotherapy [2]. The molecular events underlying the development of RILI remain poorly understood. Furthermore, effective treatments for RILI are still lacking [1]. Due to this lack of effective treatment, the prognosis for patients with RILI is poor. Fortunately, regenerative medicine provides a potential strategy to solve this problem. Since mesenchymal stem cells (MSCs) have effective immune-modulatory features, inhibit T-lymphocyte proliferation, and have a high regenerative capacity, they can play a vital role in the reconstruction of injured tissues, including bronchioles, in pulmonary diseases. A variety of studies have also illustrated the potent immune-modulatory effects of MSCs on immune lung diseases and the inflammatory response. Therefore, many scientists have attempted to treat radiation sickness with MSCs. In 2007, human bone marrow-derived MSCs (hBM-MSCs) were reported to migrate to radiation-damaged tissues in mice, providing potential evidence for restorative therapy outside of the bone marrow [3]. Since then, an increasing number of research advances have made MSCs a viable therapy for RILI; this is the focus of this review.

## RILI

**Fig. 1:**
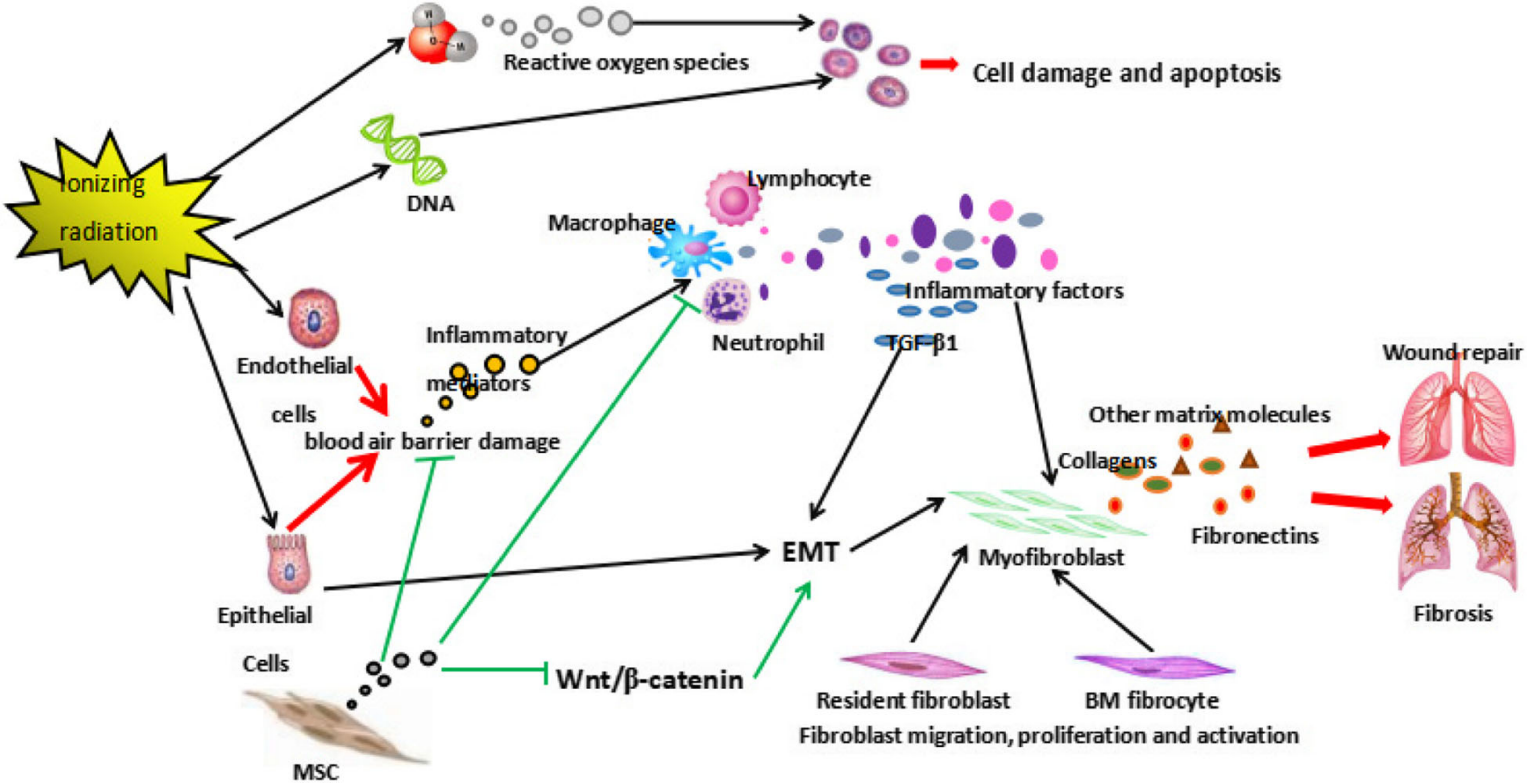
The mechanisms of radiation-induced lung injury (RILI) and the mechanisms by which mesenchymal stem cells (MSCs) alleviate it. BM bone marrow, EMT epithelial-to-mesenchymal transition, TGF transforming growth factor

When lung tissue is irradiated by x-ray cells are injured by the direct or indirect action of radiation. Once the radiation dose exceeds the radiation threshold, radiation damage may extend beyond the intrinsic repair capacity of the human body, and radiation-induced lung injury occurs (Fig. 1). RILI can be divided into two phases—radiation pneumonitis (RP) and radiation-induced pulmonary fibrosis (RIPF)—which represent acute and late phases in the development of RILI, respectively.

The exact mechanisms of RILI are largely unknown. The consensus of researchers studying RILI is that ionizing radiation could induce damage to epithelial cells and endothelial cells, dysfunction of the blood-air barrier, and increase vascular permeability. The ionizing radiation further activates alveolar macrophages and upregulates transforming growth factor (TGF)-β1, tumor necrosis factor (TNF)-α, interleukin (IL)-1β, IL-6, and IL-12 (especially the key factor TGF-β1) [4]. The immune response of human the body can amplify the process, increasing the local inflammation response and giving rise to the development of interstitial pneumonia. The persistence of chronic inflammation eventually causes lung fibrosis. In normal wound healing, this inflammation response may subside and induce a vicious cycle of further inflammation once radiation pneumonitis happens, finally leading to the poor prognosis. Alveolar epithelial cells participate in the pathogenesis of fibrosis, producing pro-inflammatory mediators, and undergoing epithelial-to-mesenchymal transition (EMT). TGF-β1, a necessary cytokine to induce EMT, promotes EMT of pulmonary epithelial cells and plays an important role in lung fibrosis [5]. In the early stage of injury, due to the chemotactic action of cytokines, bone marrow fibroblasts migrate to the lung and there promote fibrosis progression. In addition, the lung fibroblasts will also excessively proliferate after exposure to irradiation.

While a few pharmacological treatment strategies can mitigate the adverse effects of irradiation, regenerative medicine provides possible opportunities for restoring functionality to the irradiated tissue bed.

## MSCs

Among the stem cell populations, MSCs are the most extensively studied and may have the optimal outcome for regenerative medical research [6]. MSCs are commonly found in several adult tissues, including bone marrow, umbilical cord, and adipose tissue. Adipose tissue is of special interest since it represents an abundant and easily accessible source of MSCs. Friedenstein et al. first showed the existence of MSCs in the late 1960s [7]. Later, several other scholars further demonstrated that MSCs have the potential for proliferation, self-renewal, and differentiation. The superiorities of MSCs are summarized as the following five aspects [8–10]: 1) MSCs are easily obtained from multiple sources; 2) MSCs can home and engraft to injured tissues; 3) MSCs possess extensive proliferation potential and can differentiate into a wide variety of cell types; 4) MSCs have low immunogenicity and so can be transplanted successfully across immune barriers; and 5) the application of MSCs to patients has no ethical controversy.

In numerous animal studies, the physiological functions of MSCs have been well studied, especially the chemotaxis of MSCs. MSCs can selectively migrate to sites of tissue injury and exert an immunosuppressive activity by the secretion of anti-apoptotic, anti-inflammatory, and angiogenic factors, such as monocyte chemoattractant protein (MCP)-3, stromal-derived factor-1 (SDF-1), TGF-β1, vascular endothelial growth factor (VEGF), platelet derived growth factor (PDGF), and hepatocyte growth factor (HGF). This stimulates angiogenesis, building a protective environment for host cell recovery and thus preserving or even rescuing injured tissue from destruction [11–13]. Experimental studies have revealed that MSCs may have great therapeutic potential in several clinical diseases, including myocardial infarction, acute lung injury (ALI), acute respiratory distress syndrome (ARDS), and hepatic failure [14–16]. In 2012, MSC product approval was gained for the treatment of pediatric graft-versus host disease (GVHD) in New Zealand and Canada (Prochymal®; Osiris Therapeutics) [17]. Three years later in Japan, Temcell HS Injection, the same MSC product for treatment of acute GVHD, was the first allogeneic product to receive full approval [18]. In 2016, MSCs were even recommended as third-line treatment for grade II–IV acute GVHD in guidelines from the British Committee for standards in hematology [19]. With this great progress it is certain that MSC therapy will have a bright future.

## MSC-based therapy for RILI

### Feasibility of MSC-based therapy for RILI

SC therapy for lung injury specifically exists as a “first-pass” effect whereby the MSCs are expected to first reach the lung and then secret the paracrine factors needed to regulate the microenvironment [20–22]. A growing number of studies have demonstrated at least two major advantages of MSC therapy for lung injury (Fig. 1): 1) MSCs could participate in modulating the immunological response, promoting an anti-inflammatory or tolerant phenotype; and 2) MSCs could migrate to sites of injury after systemic administration, where a portion of them can save cellular injury or differentiate into the corresponding tissue cells to replace the injured lung tissue [16].

The promising clinical therapeutic effects of MSCs rely especially on paracrine and nonimmunogenic mechanisms. Although several studies have reported that injured lung tissue increases the retention of MSCs in the lungs, and that BM-MSCs are able to differentiate into airway and epithelial cells in the lung [23, 24], they will be cleared by the immune system soon after transplantation into the body. Fortunately, MSCs can secrete a variety of anti-inflammatory cytokines, such as prostaglandin E2 (PGE2), IL-10, inducible nitric oxide (iNO), and indoleamine-2,3-dioxygenase (IDO) [16]. By upregulating the expression of these cytokines, MSCs could mitigate local inflammatory injury, stimulate the proliferation of lung epithelial cells, and restrain the pulmonary epithelial cell EMT process. Additionally, BM-MSCs have been shown to release high levels of growth factors, including VEGF, which are beneficial for normal wound healing [25].

The multi-differentiation potential and the migration of MSCs to injured tissues also contributes to the possible application of MSC therapy. More recently, it has been shown that MSCs used in a mouse model of total body irradiation before transplant can proliferate without any remarkable loss in their differentiation capacities [3]. After transplantation into adult non-irradiated mice, MSCs migrate to the bone marrow or other tissues and differentiate into the corresponding tissue cells to replace the injured lung tissue [3]. Consistently, MSCs have been shown to possess a greater potential to differentiate into epithelial cells in a coculture system with irradiated lung biopsies in vitro [26]. Therefore, MSC-based therapy for radiation-induced lung injury is feasible.

### Molecular mechanisms of MSC-based therapy for RILI

The exact mechanisms of RILI are still largely unknown, so the molecular mechanisms for MSC-based therapy for RILI are still under study. The Wnt signaling pathway plays crucial roles in the differentiation and the proliferation of cells. Previously, it was demonstrated that cooperativity between the TGF-β and Wnt signaling pathways could induce alveolar epithelial cells to undergo EMT [27, 28]. Therefore, Zhang et al. further evaluated the associations between MSCs and the Wnt pathway, and found that coculture of normal human lung fibroblasts (nHLFs) with umbilical cord-derived MSCs (UC-MSCs) weakened the radiation-induced activation of Wnt/β-catenin signaling [29]. Wnt/β-catenin signaling thus became a potential therapeutic target for attenuating RILI. Then Shen and colleagues found that TGF-β1 and Wnt5a expressions correlated with the inhibition of Wnt3a/β-catenin signaling in an acute lung injury model, and that exogenously supplied MSCs could migrate to sites of lung injury and reduce the epithelial permeability, likely by blocking TGF-β1 and Wnt5a-mediated inhibition of wnt3/β-catenin signaling [30]. Xue et al. examined the paracrine influence of MSC therapy on the development of RILI [31]. Both in vivo and in vitro, MSC-conditioned medium could attenuate RILI, which indicated that paracrine activity of MSCs was very important in their beneficial effects [31]. As mentioned previously, TGF-β1 is a key factor in RILI. Huber with his team demonstrated that small-molecule inhibitors of the TGF-β receptor I kinase markedly reduced the inflammation response and pulmonary fibrosis and prolonged survival in a mouse model, which may offer a promising approach to attenuate radiation-induced lung toxicity [32].

Klein et al. first demonstrated that MSCs could protect the highly radiosensitive lung tissue from RILI in an animal model [33]. Compared with 15 Gy whole-thorax irradiation without MSC therapy, MSC therapy reduced the radiation-induced endothelial Mmp2 expression, thereby normalizing vascular function and alleviating the damage to vascular structures; it further participated in the paracrine or protective effects against lung metastasis [33].

### Animal studies of MSC-based therapy for RILI

Experimental studies have also indicated that MSCs are useful for relieving RILI [3]. Here we discuss research on RILI treated with MSCs (Table 1).

**Table 1:** Research on radiation-induced lung injury treated with MSC-based therapy

Study	Model	Cell type	Route	Efficacy results
Wei et al. 2017 [46]	Mice	UC-MSCs transduced or not to express SOD3	iv	The early treatment with UC-MSCs alone significantly reduced radiation pulmonary fibrosis, with further improvement by administration of SOD3-infected UC-MSCs
Klein et al. 2017 [47]	Mice	BM-MSCs	iv	As a MSC-secreted factor, MSC-derived SOD1 is involved in the protective action of MSCs, and it plays a pivotal role against oxidative damage
Chen et al. 2016 [45]	Mice	Mn-SOD-MSCs	iv	Mn-SOD-MSCs were successful in modulating RILI in mice
Zhang et al. 2015 [29]	Human lung fibroblasts	UC-MSCs	Cocultured	Coculture of nHLFs with HU-MSCs weakened the radiation-induced activation of Wnt/β-catenin signaling. Wnt/β-catenin signaling became a potential therapeutic target for attenuating RILI
Xia et al. 2016 [36]	Mice	BM-MSCs	iv	Low-dose hBM-MSC therapy in a dose-dependent manner better contributed to functional recovery in mice
Jiang et al. 2015 [39]	Rats	AD-MSCs	iv	AD-MSCs could relieve RILI by reducing serum levels of IL-1, IL-6, and TNF-α, increasing levels of IL-10, and downregulating TGF-β1, α-SMA, and type 1 collagen levels in irradiated lung tissues
Wang et al. 2014 [10]	Rats	UC-MSCs	iv	The UC-MSCs had definite therapeutic effects on acute radiation injury in rats
Klein et al. 2016 [33]	Mice	BM-MSCs	iv	Therapy with BM-MSCs alleviated RILI and reduced the risk of lung metastasis
Hu et al. 2013 [43]	Mice	Trx-1-overexpressing hUC-MSCs	iv	Trx-1-overexpressing hUC-MSCs prolonged the survival of injured mice
Wang et al. 2013 [42]	Mice	AD-HGF-modified MSCs	iv	AD-HGF-modified MSCs did not reduce inflammation of the lungs but inhibited fibrosis
Xue et al. 2013 [31]	Mice	AD-sTβR-MSCs	iv	MSCs and AD-sTβR-MSCs adopted the characteristics of alveolar type II (ATII) cells and significantly alleviated lung injury
Kursova et al. 2009 [34]	Mice and humans	Autologous MSCs	iv	The increasing accumulation of transplanted stem cells in the lung tissue decreased the mortality rate of mice with RILI followed by thoracic irradiation and cell therapy with MSCs did not induce progression of the underlying oncological disease in clinical trial

*AD* adipose tissue, *BM* bone marrow, *h* human, *HGF* hepatocyte growth factor, *IL* interleukin, *iv* intravenous, *Mn* manganese, *MSC* mesenchymal stem cell, *nHLF* normal human lung fibroblast, *RILI* radiation-induced lung injury, *SMA* smooth muscle actin, *SOD* superoxide dismutase, *TGF* transforming growth factor, *TNF* tumor necrosis factor, *UC* umbilical cord

After thoracic irradiation with a dose of 12 Gy in male C57BL/6 mice, Kursova et al. explored the possible effect of MSC therapy in RILI. After local thoracic irradiation, with the accumulation of transplanted stem cells in the lung tissue increased, the mortality rate of mice with RILI decreased [34].

The time window is a key point in the treatment of RILI. Yan et al. analyzed the behavior of MSCs transplanted at different time points after lung irradiation. Being controlled by the microenvironment, MSCs injected into the body immediately after irradiation helped the repair of lung injury. On the contrary, cells injected later (2 months later) were involved in fibrosis development [35].

It is unscientific to evaluate an effect if the dose factor is ignored. By observing therapeutic effects with low (1 × 10^3^ hBM-MSCs/g), medium (5 × 10^3^ hBM-MSCs/g), and high (1 × 10^4^ hBM-MSCs/g) doses in an RILI mouse model, Xia et al. found that low-dose BM-MSCs better contributed to functional recovery in mice [36].

The effect of different MSC sources may also be different. There are more studies on hBM-MSCs than UC-MSCs and adipose tissue-derived MSCs (AD-MSCs); however, the clinical use of BM-MSCs has several problems, including morbidity, pain, and low cell number on harvest [37]. UC-MSCs and AD-MSCs also have other advantages including easy expansion in vitro, a noninvasive harvest procedure, low immunogenicity, and limited ethical concerns compared with those of other stem cells. AD-MSCs have been shown to have better expansion capacity than BM-MSCs in vitro, and hUC-MSCs show faster self-renewal properties along with a painless collection procedure [37, 38]. Subsequently, Wang et al. proved that hUC-MSCs have definite therapeutic effects on acute radiation pneumonitis in rats [10]. The mouse model with acute radiation pneumonitis showed prolonged survival and reduced signs of pulmonary inflammation when animals received intravenous MSC infusions. Jiang et al. found that AD-MSCs could relieve RILI through reducing the serum levels of IL-1, IL-6, and TNF-α, increasing the levels of IL-10, and downregulating TGF-β1, α-smooth muscle actin (SMA), and type 1 collagen levels in irradiated lung tissue [39].

As mentioned earlier, by showing several benefits (especially their homing capability), the potential application of MSCs as an ideal carrier for the treatment of RILI has been well recognized. For instance, as a critical mediator of pulmonary epithelial repair, keratinocyte growth factor (KGF) could stimulate epithelial cell proliferation and repair pulmonary epithelia. James and colleagues demonstrated that gene therapy combined with stem cell transplantation delivering KGF could provide an effective therapy for bleomycin-induced pulmonary fibrosis in mice [40]. In the early years, several studies confirmed that therapeutic genes modified with MSCs could effectively transfer the target genes onto the targets, and enhance the therapeutic effects [40, 41]. All those studies provided new insight for RILI therapy due to a lack of efficacy treatments. Later, some literature indicated that MSCs as a vector could also cure RILI (Table 1).

Due to the key effect of TGF-β1 in RILI, Xue et al. combined two therapeutic strategies, MSC therapy and sTβR overexpression, and identified the curative efficacy of genetically modified MSCs for relieving RILI in a mouse model [31]. Based on the regeneration of organs being enhanced by HGF its inhibitory effect on fibrosis, Wang et al. evaluated the curative efficacy of HGF gene expression induced with MSCs on RILI, and these results proved that MSC-based HGF gene therapy does not reduce the inflammation response but also inhibits the development of radiation-induced lung fibrosis (RILF) [42]. Another candidate therapy for RILI is Trx-1, which can scavenge reactive oxygen species (ROS), adjust and control cell growth and differentiation, inhibit apoptosis, and participate in immune reactions. Furthermore, Trx-1 also decreases the excessive inflammation response in RILI by adjusting the creation of inflamed media, reducing the chemotaxis, adhesion, and migration of inflammatory cells, and inhibiting the activation of complement. In animal experiments, after irradiation with 4.5 Gy ^60^Co gamma-rays, Trx-1 overexpression induced by infusing MSCs prolonged survival compared with a control group [43]. Superoxide dismutase (SOD) plays a pivotal role against oxidative damage. A study confirmed that manganese superoxide dismutase (Mn-SOD) gene therapy could ameliorate radiation-induced intestinal syndrome in vivo [44], and thus Chen et al. attempted to research whether administration of Mn-SOD-MSCs could attenuate RILI; the results proved that Mn-SOD-MSCs were effective in relieving RILI in mice [45]. Furthermore, SOD1 as a MSC-secreted factor could mitigate radiation-induced lung endothelial cell damage and dysfunction. Moreover, through injection of UC-MSCs overexpressing SOD3, it was also found that SOD3-infected UC-MSCs more significantly reduced radiation pulmonary fibrosis, better than with UC-MSCs alone [46, 47].

In these studies, genetically modified MSC therapy also had several problems, including biosecurity issues and cell activity after gene transfection.

### Clinical research on MSC-based therapy for RILI

Although an increasing number of animal and in-vitro studies have shown good prospects for MSC-based therapy in RILI, clinical research is still lacking despite some employing MSCs in chronic lung disease [6, 48]. It has been reported that 11 patients (four breast cancer patients and seven lymphogranulomatosis patients) with RILI are being treated with MSC autotransplantation combined with standard therapy drugs, such as nonsteroidal anti-inflammatories, broncholytics and mucolytics, vitamins, antihistamine, and antibiotics drugs [34]. After 1 year of treatment, MSC-based therapy has not induced the progression of the underlying oncological disease and no adverse changes have been found for the parameters of external respiration, indexes of tissue hypoxia, or inflammatory tests and the immune state [34]. Unfortunately, these results fail to demonstrate the effectiveness of MSC-based therapy for RILI due to the lack of a control, but these data do indicate that MSC-based therapy is safe when used in the human body with radiation damage. Notably, RILF followed by asymptomatic radiation pneumonia is often diagnosed in the late stages when serious fibrosis has usually developed, and this might be difficult to effectively reverse with MSCs [49]. Therefore, potential clinical application of MSC-based therapy for RILI still has a long way to go.

## Conclusion

RILI is a major and deadly clinical complication of radiotherapy in thoracic malignancies. The quality of life and overall survival in patients with RILI is reduced. In the clinic, steroids are still the treatment mainstay for RILI. As increased academic research has produced more papers on MSCs, some scholars have attempted to treat RILI using MSCs, and have demonstrated that MSCs could alleviate radiation pneumonitis and RILF. In addition, since MSCs can migrate to the injured lungs, they can also act as cell therapy vehicles for treating RILI.

However, it is difficult to gather enough MSCs and to keep enough time of them at the injury site. MSC senescence could impair their proliferation and differentiation potential [50]. In terms of RILI, we still need to evaluate the safety of MSC therapy, and a considerable number of studies are needed to evaluate the optimal dosage, time, and route of administration. Furthermore, the therapeutic effect of MSCs in experimental animals does not always show satisfying results. Treatment effects enhanced with therapeutic genes modified by MSCs are becoming the future trend of development. Thus, seeking out a powerful therapeutic gene is a key point. In addition to this, MSCs from different sources have different characteristics. Which one is more appropriate in cell therapy? In conclusion, MSC therapy has great potential for managing RILI; however, there is still a long way to go before it will be used in clinical practice.

## Abbreviations

AD-MSC: Adipose tissue-derived mesenchymal stem cell
ALI: Acute lung injury
ARDS: Acute respiratory distress syndrome
EMT: Epithelial-to-mesenchymal transition
GVHD: Graft-versus-host disease
hBM-MSC: Human bone marrow-derived mesenchymal stem cell
HGF: Hepatocyte growth factor
IL: Interleukin
KGF: Keratinocyte growth factor
MSC: Mesenchymal stem cell
RILF: Radiation-induced lung fibrosis
RILI: Radiation-induced lung injury
RIPF: Radiation-induced pulmonary fibrosis
ROS: Reactive oxygen species
SOD: Superoxide dismutase
TGF: Transforming growth factor
TNF: Tumor necrosis factor
UC-MSC: Umbilical cord-derived mesenchymal stem cell
VEGF: Vascular endothelial growth factor
